# MotiVE BCI: motivation models including valence and expectancy in brain–computer interface use

**DOI:** 10.3389/fnhum.2026.1681683

**Published:** 2026-06-25

**Authors:** Sonja C. Kleih

**Affiliations:** Section Intervention Psychology, Department of Psychology I, Institute of Psychology, Faculty of Human Sciences, Julius-Maximilians-University of Würzburg, Würzburg, Germany

**Keywords:** brain-computer interface, motivation, P300, psychological variable, sensorimotor rhythm, theoretical framework, BCI theory

## Abstract

In this work, a theoretical framework addressing the role of motivation in brain–computer interface (BCI) was developed. The aim was to present theory-based versions of motivation models for BCI use that can serve as a foundation for hypothesis generation and experimental testing. As a synthesis of the existing literature on the role of motivation in BCI use, and grounded in a predominantly psychological theoretical background, the P300 MotiVE model and the sensorimotor rhythm (SMR) MotiVE model were introduced. To the best of my knowledge, the MotiVE models represent the first models based on psychological theories to explicitly target motivation and its subcomponents as factors influencing BCI performance. However, the underlying assumptions require empirical validation, and the practical utility of these models remains to be demonstrated. Further development may also be necessary to accommodate different types of BCI systems.

## 1 Introduction

Since the potential role of psychological factors, particularly motivation, in brain–computer interface (BCI) use was highlighted more than 20 years ago (Kübler et al., 2001), there has been growing recognition that successful BCI development must address not only technical aspects, such as improving classification algorithms, but also the human user. A model of BCI control proposed in 2011 (Kübler et al., 2011) explicitly incorporated human variables, including physiology, anatomy, and psychology. Within this framework, psychology was further divided into attention, mood, motivation, and visuomotor coordination. Among these, motivation was considered especially critical, as it might directly influence user engagement, persistence, and ultimately the effectiveness of BCI use. More recent research has reinforced the importance of user-centered factors. The acceptability of BCI systems has been shown to depend on perceived usefulness and ease of use (Grevet et al., 2023), both of which are closely tied to a user’s motivation to adopt and continue using the system. Furthermore, studies involving end users demonstrated that willingness to use BCI technology was shaped by highly individual requirements (Kübler et al., 2020). In BCIs for communication, users valued system accuracy and speed equally (Fried-Oken et al., 2024), suggesting that motivation was not only influenced by technical performance but also by how well the system aligned with user expectations and needs.

Along the same lines, qualitative research has shown that even when users reported being motivated, BCI interaction was often experienced as highly demanding (Kögel and Wolbring, 2020) and strongly influenced by contextual factors (Roc and Lotte, 2023). This suggests that motivation alone is not sufficient; rather, the willingness to engage with a BCI system might depend on the individual’s situation and their evaluation of its practical benefit. Motivation has also been discussed in relation to clinical applications, where it may interact with mood and thereby influence BCI outcomes (Vaughan et al., 2012). Extending this perspective, the concept of motivational incongruence, defined as the insufficient realization of personal goals, has been proposed as a predictor of neurofeedback success (Hernandez et al., 2018). This highlights that not only the presence of motivation but also its alignment with user goals is critical for effective BCI use.

Jeunet et al. (2017) introduced a cognitive model of BCI training that identified attention, motivation, and spatial abilities as key components of user–system interaction. All three contribute to BCI accuracy, with motivation playing a central role in sustaining engagement and supporting the effective deployment of cognitive resources. Similarly, gamification strategies have been proposed to enhance motivation by increasing immersion through game-like tasks, which, in turn, may improve attention and overall performance (Vrins et al., 2024).

Despite this growing body of work, motivation has been defined inconsistently across studies. In some cases, it referred to the willingness to use a BCI system, while in others, it has been described as the enjoyment experienced during use. This lack of conceptual clarity limits the comparability of findings and hinders theoretical progress. Therefore, a theory-driven, psychologically grounded model that explicitly defines motivation and its role in BCI performance is needed. In this work, the role of motivation from a psychological perspective was explored. First, existing findings on the effect of motivation on BCI performance were summarized. This work focused on BCIs based on sensorimotor rhythms (SMRs; see Section 1.1) and the P300 (see Section 1.2). Although a wide range of other BCI input signals exists (Valeriani and Vaughan, 2025), these two paradigms were selected because they have been most extensively studied in relation to motivational factors. Additionally, this focus allowed for a more systematic examination of how motivation influences BCI performance. As a next step, existing models of motivation were introduced. Finally, the two were linked together, and theory-driven motivation models for BCI use that are supported by existing research were presented. This endeavor will help support the categorization of the different meanings of the term motivation in original research studies and allow for theory-based BCI research in clinical and healthy participants.

### 1.1 BCI studies based on sensorimotor rhythms investigating motivation

BCI systems based on motor imagery use the fact that SMRs in the alpha band from 8 to 12 Hz (*μ* rhythm) and the beta band from 18 to 25 Hz over the sensorimotor cortex (SMC; M1/S1; Pfurtscheller and Neuper, 2010) desynchronize, with a decrease in amplitude (event-related desynchronization = ERD) as a result of motor imagery. This desynchronization can be classified by the BCI system. Given that SMR-BCI control constitutes a learning task, the effect of motivation on self-regulated learning can be hypothesized to be particularly pronounced. For clarification of this hypothesis and synthesis of the literature a review on SMR-BCI studies that have either monitored or experimentally manipluated motivation is required.

Nijboer et al. (2008) trained 16 healthy participants to regulate their SMR for BCI control. In one group, feedback was visual, while in the other group, it was auditory. In the auditory condition, the sounds of bongos represented ERD. Motivation was investigated using the Questionnaire for the Assessment of Current Motivation during BCI use (QCM-BCI, Nijboer et al., 2008b). The QCM-BCI is a slightly modified version of the original QCM (Rheinberg et al., 2001) to account for the use of a BCI instead of making predictions about performance and success in cognitive learning tasks. In the visual feedback condition, the authors found a positive relation between mastery confidence and performance and a negative relation between incompetence fear and performance. Interestingly, in the auditory feedback condition, only the subscale of incompetence fear was positively related to performance. The authors assumed that visual feedback was easier than auditory feedback, which is why, in the auditory condition, high fear of incompetence might have boosted performance and supported attention allocation to the target stimuli, which was unnecessary in the easier visual condition. Kleih et al. (2013) also identified an influence of incompetence fear on performance but found a negative relationship between incompetence fear and BCI performance in a visual SMR paradigm study. This indicates that the effect of motivation-related factors may depend strongly on task characteristics and context. Besides the effect of incompetence fear, in their sample of 51 healthy participants and 11 post-stroke patients, healthy individuals demonstrated a positive correlation between the motivation component of interest and performance, while stroke patients showed positive associations between experienced challenge and BCI performance, and mastery confidence and BCI performance.

Duan et al. (2023) demonstrated that the motivational aspect of challenge can be enhanced through feedback design. They introduced an approach incorporating performance data from past trials into the visual feedback of the current trial. This additional information yielded improved class distinction in SMR-BCI performance for the group receiving transfer learning feedback compared with the control group, which received standard feedback containing only current-trial information. This finding supports the call for novel BCI protocol designs that enhance user engagement (Chavarriaga et al., 2017; Jeunet et al., 2018).

In another study (Kleih-Dahms et al., 2021), the effect of monetary reward on motivation and BCI performance was investigated in healthy participants. Participants were assigned to either a motivated group, who received additional trial-based rewards contingent on their SMR-BCI performance, or an unmotivated group, who could not earn extra rewards. The motivated group showed higher r^2^ values compared with the unmotivated group. However, although motivated participants outperformed the unmotivated group in terms of the percentage of correctly classified trials, this difference was not statistically significant.

Motivation in SMR-BCI use has also been investigated as a complete construct rather than examining individual components. In an SMR-BCI-based rehabilitation study involving patients with upper limb motor impairments following stroke, Morone et al. (2015) found a significant correlation between self-reported motivation (measured via visual analog scale) and BCI performance during hand motor imagery. The authors hypothesized that motivation constitutes an essential component of BCI-based rehabilitation protocols.

However, not all studies investigating the potential role of motivation in BCI performance have reported such an effect. In a study with four patients with amyotrophic lateral sclerosis (ALS), no correlation between motivation ratings and BCI performance was found (Pitt and Brumberg, 2022). In a study by Hammer et al. (2012), no influence of mood or motivation on SMR-BCI performance was found. Among all the tested variables, the authors found that performance in a two-hand coordination test explained 11% of the variance in SMR-BCI performance. The outcome variable “performance level” of the Attitudes toward Work test (Arbeitshaltungen-Analyse (AHA), Kubinger and Ebenhöh, 1996) can be interpreted as an indicator of how well a participant was able to focus on the BCI task and accounted for approximately 19% of the variance in SMR-BCI performance.

### 1.2 BCI studies based on the P300 event-related potential investigating motivation

The event-related potential (ERP) P300 is a positive electrical deflection that occurs approximately 200–600 ms after the perception of a rare target stimulus, which is presented among a stream of frequent, non-target stimuli, known as the oddball paradigm (Sutton et al., 1965). In a BCI setting for communication, the user focuses attention on a specific letter within a matrix of letters arranged in rows and columns. Each row and column flashes in random order. By mentally counting only the flashes of the desired letter, a P300 is elicited. The BCI system detects the P300 and classifies it to identify the target letter (Farwell and Donchin, 1988). In the context of motivation, it is highly probable that its influence manifests through the modulation of attentional allocation toward the task, subsequently affecting P300 amplitude. Relevant studies are reviewed below to summarize existing findings on this topic.

The first experimental research on the topic was performed by Kleih et al. (2010), who experimentally manipulated motivation using monetary reward in a BCI study. The authors offered participants 0, 25, or 50 € cents per correctly spelled letter but did not find a relation between the monetary reward and BCI performance. However, when using the motivation values assessed before motivation manipulation with a visual analog scale (VAS) ranging from 0 (not motivated at all) to 10 (extremely motivated), a positive correlation between the VAS values and the P300 amplitude at Cz was found. In addition, intrinsic motivation was explored by providing healthy participants with information about BCI research and the potential benefits of their participation for BCI end users. Contrary to the hypothesis, this motivational intervention had no measurable effect on P300-BCI performance (Kleih and Kübler, 2013). Subsequent analysis of perspective-taking ability as a potential moderating variable revealed an unexpected finding: participants with lower perspective-taking capacities demonstrated higher P300 amplitudes. It may be that lower emotional involvement allowed for higher attentional allocation to the BCI task. Based on these results, it was hypothesized that high emotional involvement may detrimentally affect BCI performance by competing for attentional resources, thereby reducing focus on task-relevant stimuli despite increased motivational engagement (Kleih and Kübler, 2013).

Käthner et al. (2013) investigated 20 healthy participants with an auditory and visual P300 speller and assessed motivation with a VAS. The authors reported a correlation between the values in a VAS for motivation measurement and performance in an auditory BCI, with motivation explaining 19% of the variance in the P300 amplitude at Pz. Interestingly, this influence of motivation only appeared in the auditory condition, in which average accuracy was 66%, compared with the visual condition, in which average accuracy was 94%. It seems that only in the more difficult paradigm, as indicated by a higher subjectively reported workload (Käthner et al., 2013), motivation affected performance. This finding was supported by Baykara et al. (2016), who found a clear correlation between motivation and performance in an auditory BCI spelling paradigm in healthy students. When Hammer et al. (2018) investigated psychological variables influencing BCI performance, they did not find a direct relationship between motivational components and performance outcomes. However, they reported that learning ability was positively correlated with performance in a visual P300 BCI. Motivation may influence BCI performance indirectly, for example, by supporting learning processes that might also play a role in ERP BCI use.

In a study by Nijboer et al. (2010), it was investigated whether motivation would influence P300 BCI performance in participants with ALS. In two of six ALS participants, the motivational components challenge and mastery confidence were positively related to BCI performance, whereas incompetence fear was found to negatively influence performance in one participant when using the P300 BCI. Supporting these findings, another study involving ALS participants using the P300 speller demonstrated a positive correlation between mastery confidence and spelling speed (Kleih and Kübler, 2014). However, the provision of gift certificates as monetary incentives failed to produce a significant impact on either motivation or BCI performance.

Another, more indirect approach to studying motivation is through gamification. Although incorporating game elements into BCI protocols does not automatically enhance user motivation, this effect has frequently been reported in the literature.

### 1.3 BCI studies investigating motivation through gamification and virtual reality experience

For several years, the potential benefits of gamified BCI feedback (for a review, see Ahmed et al., 2025) and the use of virtual reality (VR) (Berger et al., 2022) have been extensively investigated. Both approaches aim to increase immersion and user involvement (Gao et al., 2022) and, ultimately, enhance motivation (Kober et al., 2024). Additionally, it has been suggested that integrating VR techniques into BCI protocols for neurorehabilitation may positively influence neural plasticity by simultaneously activating multiple perceptual channels (Borisova et al., 2023). Empirical studies provide mixed but promising evidence. For example, in an SMR-BCI study, Mladenović et al. (2017) demonstrated the beneficial effects of gamified feedback in offline performance analyses. Instead of conventional arrow cues indicating left- or right-hand motor imagery, participants controlled a penguin navigating through snow to catch fish. Similarly, Berger et al. (2022) compared 2D and augmented reality (AR) neurofeedback and found that healthy participants in the AR condition reported a tendency toward higher flow singular experience. In contrast, the 2D group reported higher technology-related fear and lower perceived usability.

Positive effects of gamification have also been observed in clinical populations. In a rehabilitation study with stroke patients suffering from upper limb paralysis, de Castro-Cros et al. (2020) reported high satisfaction with a gamified feedback system in which participants controlled a mouse to catch pieces of cheese based on motor imagery performance (Recoverix® feedback, based on Morone et al., 2015). However, findings regarding more immersive 3D feedback are less consistent. Kober et al. (2016) found that stroke patients experienced higher incompetence fear and lower mastery confidence when trained with 3D neurofeedback than with a 2D paradigm. In contrast, this negative effect was not observed in healthy older participants, where motivation values were descriptively higher for 3D than for 2D feedback (Kober et al., 2017). These results suggest that 3D feedback may be more challenging and potentially too complex for neurological patients.

Finally, individual differences such as age appear to play an important role. Kober et al. (2024) highlighted that older individuals may be less familiar with 3D technologies and more skeptical of their use. Moreover, cybersickness tends to occur more frequently in individuals aged 40 and above, which may further hinder the implementation of 3D- or AR-based feedback systems.

Collectively, these findings indicate that while gamification and immersive technologies hold promise, the assumption that gamified design inherently boosts motivation remains largely untested. Consequently, the relationship between motivation (or its components) and BCI performance (Kadosh and Staunton, 2019) remains unclear and warrants further, more targeted investigation.

A critical barrier currently hindering progress in the investigation of the effect of motivation on BCI performance is the absence of a theoretical framework to systematically examine motivation’s influence on BCI performance. Future hypotheses should be grounded in such a framework to allow for logical derivation and empirical testing. The outcomes of this research could validate the framework, necessitate its refinement, or lead to its rejection if it proves inadequate. Establishing a solid theoretical foundation is, therefore, the essential first step in enabling this iterative process of theory testing and development.

## 2 Theories on motivation in a BCI context

For a BCI end user, whether a healthy student participant or a person with a neurological or degenerative disease, there must be some driving force, some kind of motivation for participation in a BCI study or project, as BCI systems are not easily accessible and typically require an enrollment process before use. One definition that might specify this force was suggested by Reeve (2008):

*The study of motivation concerns those processes that give behavior its energy and direction. Energy implies that behavior has strength – that it is relatively strong, intense and persistent. Direction implies that behavior has purpose – that it is aimed or guided toward achieving some particular goal or outcome* (Reeve, 2008, p.8, l. 18-22).

Another possible definition was provided by Rheinberg and Vollmeyer (2012):

*The thing it (motivation) is about, is that 1) a person follows a goal, 2) is willing to make an effort and 3) stays at the task without distraction* (Rheinberg and Vollmeyer, 2012, p. 14, l. 10-12).

From these definitions, it can be deduced that motivation allows individuals to pursue a specific goal. This also means that there must be some kind of expectation that a certain goal can be achieved and that engaging in a particular action will lead to this goal. In a BCI context, this implies that a participant expects learning how to use the BCI system to be worth the effort to achieve BCI control in the end. BCI control, per se, therefore, holds a certain value for the user. This function of individuals’ expectancies and the value they assign to outcomes is described by expectancy–value theories, which could provide a useful framework for modeling motivation in BCIs.

### 2.1 Expectancy × value theories

All expectancy × value theories share the principle that motivation to initiate or sustain a given action is determined by two core components: the subjective expectation that the action will lead to a particular outcome, and the perceived value or consequence of achieving that outcome (Feather, 1982). These two components are multiplicatively related. Therefore, the basic equation in all expectancy × value theories is presented in Equation 1:


Expectancyxvalue=motivation tendency (1)

Some important examples of expectancy × value theories are Lewin’s Field Theory (Lewin and Cartwright, 1963), Atkinson’s Achievement Motivation Theory (Atkinson, 1957), Rotter’s Social Learning Theory (Rotter, 1954), and Vroom’s Instrumentality Theory (Vroom, 1964). As implied by Equation 1, motivation can be increased by either increasing the expectation component or increasing the value component, as a change in either component can affect the level of motivation.

The problem in transferring expectancy × value theories to BCI research is that individuals typically already have an idea of what consequences to expect from a certain action. This is the reason why a person can develop an expectation of how likely an action–outcome will occur. In a BCI setting, participants are often naïve. Therefore, even though a person will most likely believe in being able to acquire BCI control and thus use the BCI system, no prior experience allows for clear anticipation of this expectation. The value in a BCI context would be gaining BCI control. However, the valence of this ability might vary across individuals and also depend on further consequences, such as monetary reward or communication independence. Keeping in mind these prerequisites for the application of expectancy × value theories in a BCI context, the Instrumentality Theory by Vroom (1964), which was traditionally applied to organizational psychology (Isaac et al., 2001; Mitchell and Albright, 1972), will be introduced.

Vroom’s Instrumentality Theory (1964) builds on the work of Peak (1955), who introduced the term instrumentality as the expectation that situational outcomes lead to desirable outcome consequences. In an organizational psychology context, this would translate, for example, into the achievement of a work-related task and the receipt of a bonus payment in return. In Instrumentality Theory (Vroom, 1964), three models were combined: the Valence Model, the Action Model, and the Performance Model.

In the Valence Model, the term valence was defined as the affective orientation toward outcomes (Vroom, 1964). Valence ranges between −1 and 1 and is positive when a person prefers to attain a certain outcome rather than not attaining it. A valence of zero indicates indifference toward outcomes, while negative valence reflects a person’s preference not to attain an outcome compared to attaining it. Valence was, in contrast to value, defined as the anticipated satisfaction from an outcome, whereas value refers to the actual satisfaction evoked by the outcome. According to the Valence Model (Vroom, 1964), the valence (V) of an outcome j can be determined by the sum of the products of all valences of outcome consequences k (Vk) with the corresponding instrumentalities (I) of the outcome j causing the outcome consequences k. Therefore, the valence of an outcome j can be expressed by Equation 2:


$$\mathrm{Vj}=f\;[\sum_{k=1}^{n}(\mathrm{Vk}*\mathrm{Ijk})]$$ (2)

When transferring the Valence Model to a BCI context, the valence of BCI control as an outcome would probably be positive. Outcome–consequence k could include monetary reward for participation or independent use of the system, and instrumentality would be represented by mastering BCI control in the experimental blocks to receive an overall monetary reward or to achieve further BCI control. Therefore, the valence of BCI control is a function of the multiplied valence of gaining BCI control and receiving the reward. It is possible that monetary reward or BCI control has high valence for a person, even though participation in the study itself may have low valence for the participant. In this example, it becomes obvious that the BCI context is a relatively well-defined field compared to the organizational psychology context, in which there are many influencing factors that determine, for example, occupational choice or work satisfaction (Vroom, 1964). Moreover, the nature of valence is likely to differ in a BCI context compared to a traditional work context. It can be proposed that two different qualities of valence can be defined in a BCI context: (1) inherent valence (Vin, using the BCI system) and (2) valence by reward (VbR). Inherent valence represents the valence that originates from the task of using a BCI itself, while valence by reward refers to the valence of a monetary or other external reward associated with BCI use.

The Action Model shows how, among several possible action choices, one is preferred over another and why this one is preferred. In a BCI context, this allows for the examination of why volunteers decide to participate in a BCI study rather than in a questionnaire study. In the Action Model, expectancy is added as a factor. Expectancy was defined as a person’s subjective belief, ranging from 0 to 1, that a specific action will lead to a specific outcome. Expectancy is maximally high when a person believes that an action will certainly lead to an outcome. If a person believes that an action will not lead to an outcome, expectancy is zero. The Action Model postulates (Vroom, 1964) that the motivation with which an action is pursued, also called force (Fi), is determined by the sum of the products of the expectancy (E) to reach a certain action result j by performing action i and the valence (V) of the outcome j. Equation 3 represents this relationship:


$$\mathrm{Fi}=f\;[\sum_{j=1}^{n}(\mathrm{Eij}*\mathrm{Vj})]$$ (3)

Transferring the Action Model to a BCI context leads to the assumption that a person’s motivation, the force (F) associated with participation in a BCI study (Fᵢ), increases with both the belief (E) that the action of participation (i) will lead to a particular reward (j), such as compensation or BCI control, and the valence (V) of that reward (j). The expectancy component would, in a BCI context, probably be best represented by mastery confidence (Nijboer et al., 2008, 2010; Kleih and Kübler, 2014). Thus, the expectancy component seems applicable in a BCI context.

Vroom (1964) also postulated the Performance Model, in which he explains why performance is not solely dependent on motivation but is also influenced by ability. An action result j is determined by the product of the motivation for an action i (force = Fi) and the ability to achieve an action result (ability j, see Equation 4a):


Action resultj=(Fi∗abilityj) (4a)

As Fi can also be expressed by the relationship between Eij and Vi (see Equation 3), the action result can also be expressed accordingly (see Equation 4b):


$$\text{Action result}\;j=f(\text{ability}\;j)*[\sum_{j=1}^{n}(\mathrm{Eij}*\mathrm{Vj})]$$ (4b)

**Figure 1:**
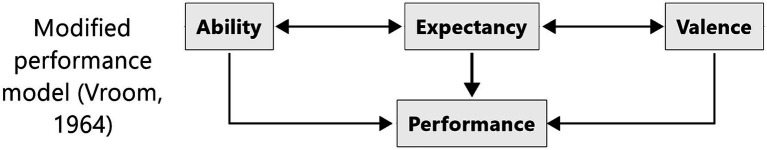
Basic BCI motivation model.

In a BCI context, the level of BCI control, therefore, depends on the BCI end user’s motivation to gain BCI control or to receive a reward and on the ability to use the BCI system, such as being able to use motor imagery for BCI control (by operant learning) or focusing attention on a P300 BCI task (Kögel and Wolbring, 2020). For a BCI context, the basic motivation model, therefore, can be formulated as depicted in Figure 1.

Within this basic motivation model, one specificity concerning valence is that it cannot be negative. Negative valence would mean that a BCI user would rather not experience outcome consequences of BCI control or would attempt to avoid them. If this was the case, this person would not choose to participate in a BCI study, as participation in research experiments is voluntary. Therefore, valence ranges between 0 and 1 in a BCI context instead of between −1 and 1 as in the original model. Furthermore, inherent valence and valence by reward are very likely independent. For example, in a reward-sensitive person, Vin could be zero, while VbR could be maximal, whereas in an ALS patient, it is very likely the opposite. Even if a patient was offered a reward, which would probably be appreciated, valence by reward might not change at all or increase only slightly, while Vin would remain high (Kleih and Kübler, 2014). Therefore, in Equation 5, it is proposed to focus on the dominant valence, as it is most likely to influence behavior, while disregarding less distinct valence components, which are expected to contribute little or not at all to the primary valence. This circumstance is mathematically expressed as max (Vin, VbR) to indicate that the dominant valence should be considered for the determination of motivation strength (see Equation 5).


$$\begin{array}{l} \text{Action result BCIc}=f(\text{ability BCIc})*\\ \left[\sum_{j=1}^{n}(E\;\text{rbrBCIc}*\max(\mathrm{Vin},\mathrm{VbR})\right] \end{array}$$ (5)

Remarks. BCIc = BCI control, rbr = regulation of brain response, E = expectancy, Vin = inherent valence by the task itself, VbR = valence by reward.

In a BCI context, the sum of the products of expectancy and valence can be operationalized through repeated measurements over time. Assessing expectancy and valence, e.g., at the start of the experiment and again after the first, second, and subsequent blocks, may reveal changes in the strength of the motivational component. For instance, the expectancy that focused attention will lead to BCI control may evolve with experience using the system. Similarly, the valence of outcome consequences for later training blocks may shift as participants gain insight into what using a BCI entails. Multiple assessments of these components thus allow for tracking changes in motivation over time, and, when combined with the ability component, can provide a more valid estimate of motivation’s influence on BCI performance.

The aforementioned distinction between valence by reward and inherent valence is based on another important concept, namely the distinction between extrinsic and intrinsic motivation. Intrinsic motivation is understood as a form of motivation in which one performs a task because of the task itself and interest in the task (Deci and Ryan, 1985; Hidi, 2000; Ryan and Deci, 2000a). Motivation is considered intrinsic when an action is sustained by the spontaneous pursuit of optimal challenge within the activity (Reeve, 2008). The activity is perceived as enjoyable for the individual engaged in it and might even cause a person to get immersed in a task, which can be described as a flow experience (Csikszentmihalyi and Csikszentmihalyi, 1992). The action is performed solely because of its inherent characteristics, rather than to achieve a subsequent goal or future outcome.

In contrast, extrinsic motivation is understood as motivation that is driven by the anticipated consequence of a certain action, which might be rewards, incentives, praise, or privileges (Deci and Ryan, 1985; Hidi, 2000; Sansone and Smith, 2000). When an action is taken based on extrinsic motivation, the same action would not have been taken without the anticipated action consequence, which is perceived as rewarding (Reeve, 2008; Rheinberg and Vollmeyer, 2012).

Since both intrinsic and extrinsic motivation are likely to play a major role in BCI use, depending on the user’s goals, theories of intrinsic and extrinsic motivation should be elaborated and integrated into a BCI motivation model where appropriate.

### 2.2 The cognitive evaluation theory

The Cognitive Evaluation Theory (CET, Deci and Ryan, 1985) covers social and environmental mechanisms influencing intrinsic motivation. Within CET, Deci and Ryan (1985) postulate three components that enable a person to act with intrinsic motivation and experience autonomy and competence. These three components are as follows: (1) the need for autonomy, (2) the need for competence, and (3) the need for relatedness. These three components are influenced by (a) action aspects and (b) intrapersonal aspects.

Deci and Ryan (1985) emphasized a person’s need to act autonomously, meaning that a person should have the feeling of being able to decide whether to initiate an action based on their own will. Actions are mostly experienced as autonomous when they are in concordance with personal values and beliefs and are not determined entirely by external situational factors. The concept of perceived autonomy unites the concepts of (1) internal perceived locus of causality (PLOC; Heider, 1958), (2) volition, and (3) perceived choice over one’s actions. PLOC can be internal or external. PLOC is internal when actions are initiated by the individual as the source. In contrast, PLOC is external when the reason for an action is to avoid the consequences of not taking an action, to fulfill a duty, or to meet another person’s expectation (Heider, 1958). Burde and Blankertz (2006) found that internal PLOC was positively correlated with performance in SMR-BCI, which suggests the importance of this concept in BCI use.

Volition can be defined as the unpressured willingness to engage in a task without feeling coerced to do so (Ryan et al., 2008). Another concept inherent in perceived autonomy is perceived choice over one’s actions. Perceived choice over one’s actions allows individuals to follow self-determined decision-making processes and actions. Based on this concept, the worst situation would be one in which no choices can be made. When different alternatives are available, perceived autonomy increases; however, only when there is truly free choice can autonomy be fully experienced (Deci and Ryan, 1985).

The second major component of CET is the need to feel competent. However, simply experiencing competence does not strengthen intrinsic motivation; PLOC must be internal (deCharms et al., 2005; Fisher, 1978). Competence arises from mastering optimally challenging tasks that are neither too difficult, which can cause anxiety or frustration, nor too easy, which may induce boredom. Thus, tasks should be matched to a person’s skill level. Feedback is critical for helping individuals understand which behaviors lead to task mastery and which do not (Ryan and Deci, 2000b). The earlier and more precise the feedback during the learning process, the more effectively a person can use it. Negative feedback can diminish motivation, whereas positive feedback reinforces the sense of competence, either by encouraging engagement in the task itself or by allowing performance comparisons with past achievements or with others (Deci, 1975).

The need for relatedness is the third major component of CET. This component is based on the observation that children who are securely attached to their parents (Bowlby, 1977) show higher levels of exploration and curiosity, reflecting greater intrinsic motivation to engage with their environment, a pattern that persists into adulthood. Ryan and Grolnick (1986) found that students were less motivated when they perceived their teachers as uncaring or emotionally distant. Thus, a sense of security and relatedness appears important for the development of intrinsic motivation, even though many intrinsically motivated activities can be performed independently of others.

CET components are influenced by action aspects and intrapersonal aspects, meaning that the ability to experience autonomy, competence, and relatedness is dependent on the aspects of an action and a person’s subjective judgments about that action. Actions include three aspects: informational, controlling, and amotivating aspects. Depending on the level to which these components are activated in the person’s perception, they can increase or decrease intrinsic motivation. Informative aspects increase motivation (e.g., a BCI user is told the accuracy with which an individual can control the BCI), while controlling aspects, which may induce pressure, decrease motivation (e.g., when a BCI user is told that only the top five performers will be able to participate in BCI studies again). Controlling aspects force a person into a predefined action, preventing free choice of action. The act of enforcement itself may be realized through social influence techniques for behavior manipulation or may even create feelings of guilt or threaten self-esteem in the other person. Amotivating aspects convey the message of incompetence, thereby hampering enjoyment in the task (e.g., when a BCI user is told that they did not correctly select a single letter). The judgment of whether an event is perceived as controlling, informational, or amotivating may depend strongly on the individual. Therefore, Deci and Ryan (1985) included another component in CET: intrapersonal aspects. The intrapersonal aspects component comprises the same aspects as the action aspects (informative, controlling, and amotivating), but focuses on the subjective judgment of the person. The assumption of intrapersonal aspects is crucial, as the motivation level is determined solely by the individual, without taking “objective criteria” into account. For example, consider an ALS patient who expresses a desire to participate in a BCI study. If the patient’s decision is entirely self-directed, it represents an autonomous choice. However, it is also possible that the patient’s family or caregivers encourage or even pressure the patient to enroll in a BCI study, seeking to explore further ways of interaction, while the patient might be satisfied with an eye-tracking system. In this case, participation reflects a controlling influence. Conversely, if the patient, in addition to experiencing external pressure, recognizes the potential for increased communication and independence, this represents an informational intrapersonal factor that could enhance intrinsic motivation.

**Figure 2:**
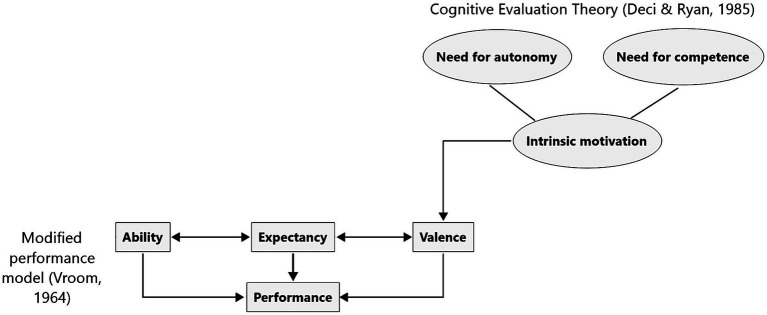
BCI motivation model including intrinsic motivation.

CET provides a motivation framework applicable to ALS patients or other clinical end users, for whom interest in using a BCI system is often driven by strong intrinsic needs for autonomy and competence. Using a BCI for communication may bring joy and satisfaction while also fulfilling the need for relatedness. Therefore, these factors were incorporated into the motivation model (see Figure 2). In contrast, healthy participants might have other opportunities to satisfy their needs for autonomy and competence beyond BCI use. For these participants, extrinsic factors may play a more significant role, as accounted for in Deci and Ryan’s Self-Determination Theory (SDT) (Ryan and Deci, 2000a).

### 2.3 The self-determination theory

In their SDT, Ryan and Deci (2000a) build on the autonomy component of CET (Deci and Ryan, 1985) and propose a continuum of self-determined behavior, ranging from non-self-determined to fully self-determined. Behaviors along this continuum differ in motivation levels, self-regulatory styles, PLOC, and associated regulatory processes. When an action lacks self-determination entirely, amotivation occurs: the individual either does not act or performs the action without pursuing any internal goal. Amotivation may arise from undervaluing an activity (Ryan and Deci, 1995) or from feeling incompetent to handle a task (Ryan and Deci, 2000a). At the opposite end of the continuum, fully self-determined behavior reflects intrinsic motivation, characterized by interest in and enjoyment of the activity itself. Between these extremes, Ryan and Deci (2000a) identify four levels of extrinsic motivation, differentiated by the degree of autonomy they permit.

External regulation provides no autonomy; behavior is performed solely to obtain an anticipated outcome, such as a monetary reward. The presence or absence of this consequence determines the action, which is why PLOC is external. When self-control is present even to a limited extent, there is a higher level of autonomy, and the person adheres to internalized norms of how to behave, which is referred to as introjected regulation (Ryan and Deci, 2000a). In introjected regulation, a person acts in accordance with what they believe others expect, primarily to avoid feelings of guilt or anxiety. PLOC is external, and the reasons for behavior are not perceived as truly belonging to oneself. At the next level of self-determined behavior, a person acts because the behavior is personally valued and considered important. Compared to introjected regulation, autonomy is higher, and this is referred to as identified regulation. In integrated regulation, the outcomes of a behavior are so aligned with a person’s values that people willingly engage in the task. In this study, behavior stems from the genuine belief that the action reflects their own convictions and is what they truly ought to do. Integrated regulation is the fourth level of self-determination and the stage where motivation is still extrinsic, but PLOC changes to being clearly internal compared to the three lower levels of self-determination and their corresponding extrinsic motivation (external, introjected, and identified regulation), where PLOC ranges from external to nearly internal. The highest level of self-determination is intrinsic regulation, in which motivation and PLOC are fully internal, and behavior is driven by personal interest and enjoyment.

Ryan and Deci (2000b) noted that the integration of extrinsic motivation can be supported by enhancing the perception of competence, autonomy, and relatedness. Interestingly, Deci et al. (1994) found that in a simple reaction-time experiment in which participants pressed a button in response to a light, providing a rationale for the task (e.g., “This task is intended to improve concentration”), acknowledging participants’ feelings (e.g., “I understand this task might be boring”), and minimizing controlling instructions led participants to spend more time on the task. Moreover, the level of internalization and integration of the task was higher compared with conditions in which these strategies were not used. These results are highly relevant for BCI research. First, participants might initially participate in a BCI study because they are aiming to receive a reward. However, after the first contact (e.g., via phone), they are introduced to the basics of BCI research. As BCI research was developed to support paralyzed people, participants’ need for relatedness could be activated. Participants could feel like contributors to something they personally value, which closely approximates the process of internalization. Before starting the BCI experiment, participants are typically informed that they can interrupt the measurement any time without providing reasons and that they can leave the experiment if they wish, which, together with the experimenter being spatially separated, contributes to the feeling of autonomy. As performance in BCI experiments is fed back to the user at the latest after one run (in P300 experiments) but continuously with every trial in SMR-BCI use, the need for competence is fulfilled as knowledge of results allows users to precisely evaluate their ability to master the BCI task.

**Figure 3:**
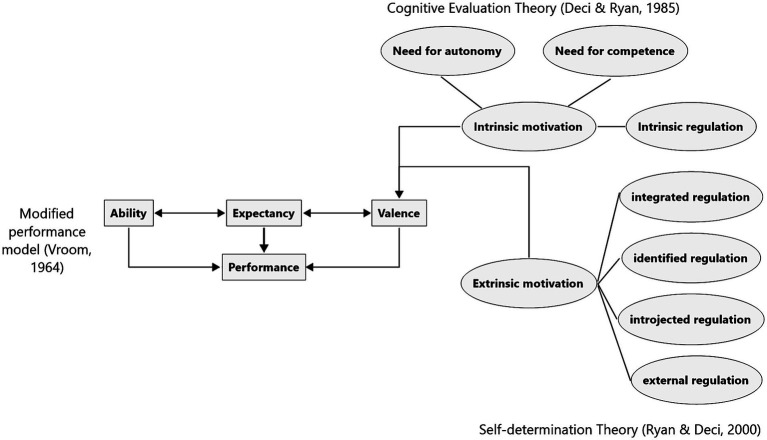
BCI motivation model including intrinsic and extrinsic motivation.

In conclusion, It can be assumed that BCI research in healthy volunteers is an area in which an originally only externally regulated behavior (i.e., participating to receive a reward) could change into an internalized or even integrated regulation driven by individual interest. Not only are most BCI users interested once they understand the topic, but they may also become engaged in supporting research for a clinical population, which may be congruent with their own values. However, it is also possible that motivation remains extrinsic and that participation is based solely on the anticipated reward (see Figure 3).

Besides the distinction between extrinsic and intrinsic motivation, it is also important to consider that any BCI end user, whether a patient or a healthy student, interacts with the BCI system within a specific environment. This environment may range from a living room or bedside setting, potentially in the presence of family members, to a BCI laboratory or an electroencephalography (EEG) cabin that may be confined and dimly lit. The person × situation interaction may affect motivation and, therefore, should also be considered.

### 2.4 Learning motivation and the person–situation interaction

Rheinberg et al. (2000) postulated in their Model of Self-Regulated Learning four steps leading to the achievement of goal-directed behavior. The first step is goal setting and/or determination of perceived incentives, which directly influence the second step, the strength and quality of the motivation that is directed toward an action (Rheinberg et al., 2000; Hidi, 2000). This motivation, in turn, influences the third step, the persistence with which the necessary actions for goal achievement are sustained, for example, the level of activity with which a person performs an action (e.g., alertness vs. tiredness). Another characteristic indicating persistence is the time spent on the task, representing the eagerness with which a person expends effort toward goal achievement (Rheinberg et al., 2000). This third “persistence step” influences the fourth and final step, which leads to the achievement of goal-directed behavior or action, resulting in a certain level of performance and quality of outcome. Rheinberg et al. (2000) furthermore postulated the necessity of a basic initial level of motivation for goal-directed behavior, which influences all presented steps and supports action initiation and adherence to goal achievement.

**Figure 4:**
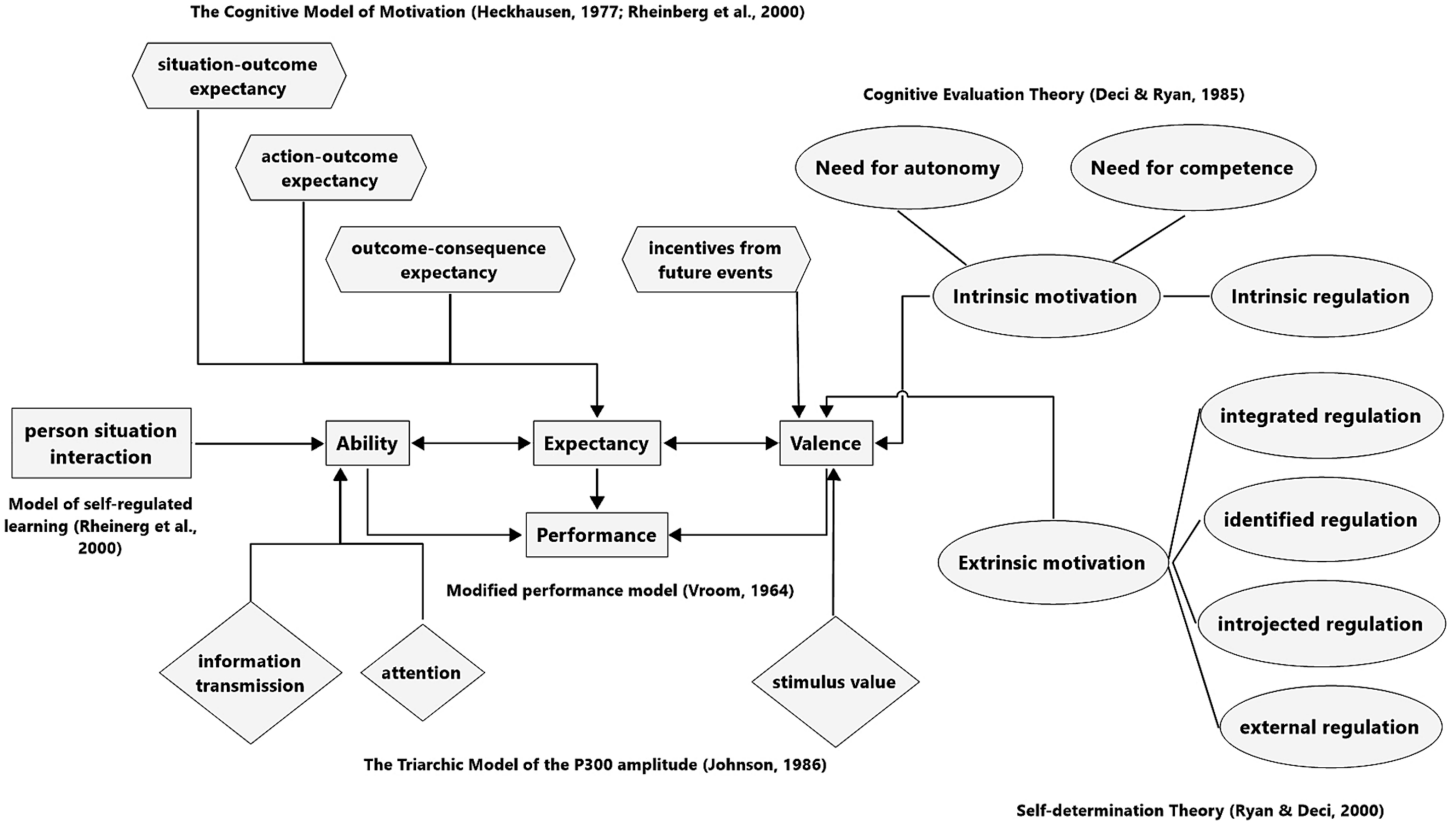
Integrated theoretical framework on the influence of motivation in BCI control.

Motivation and behavior are influenced by both personal and situational characteristics, which interact dynamically. From a personal perspective, factors such as personality, educational background, interests, and individual needs shape a person’s goals and their perception of incentives, thereby affecting all subsequent steps toward goal attainment. Situational factors also play a critical role. A task necessary for achieving a goal may be perceived as overly complex or challenging, potentially lowering goal setting and motivation due to the belief that the task is unachievable. Conversely, if task difficulty is perceived as well-matched to the person’s abilities, motivation and goal-directed behavior may be enhanced (Rheinberg et al., 2000). Given that the person–situation interaction may affect the ability to use a BCI system, it has been incorporated into the BCI motivation model (see Figure 4).

In summary, an integrative framework explaining why and how motivation influences BCI use has been developed (see Figure 4). What remains to be addressed in this model are the mechanisms by which motivation specifically affects BCI input signals, such as SMR and P300.

### 2.5 How motivation influences SMR-BCI performance: insights from the cognitive model of motivation

SMR-BCI control is based on self-regulated learning. Therefore, the integration of a model that addresses this self-regulatory aspect is necessary. In the original model, Heckhausen (1977) proposed the perceived situation to be the basis on which a learner decides to take an action, driven by the belief of being able to master the action or actions necessary for the achievement of an intended goal or outcome. For action completion, Heckhausen (1977) postulated different kinds of expectancies, through which the stages from a perceived situation to consequence anticipation are connected. First, a person determines whether an action is required or whether the situation itself determines the outcome, which is referred to as situation–outcome expectancy. In other words, situation–outcome expectancy (SOE) defines the likelihood with which a desired outcome might occur without any input from the actor. If a person believes that the situation itself does not already determine the subsequent outcome, action–outcome expectancy (AOE) represents the next step, reflecting whether an action can influence the outcome. The actors’ ability or effort is required and has the potential to influence the outcome. When a person develops AOE, the incentive value of potential consequences must be sufficient to sustain purposeful actions. Both SOE and AOE range between 0 and 1 (Heckhausen, 1977). Finally, outcome–consequence expectancy (OCE) determines whether a desired consequence is to be expected from the outcome. OCE defines the degree to which outcomes lead to certain consequences that have valence for the actor. OCE ranges between −1 and 1 (Heckhausen, 1977).

Heckhausen’s (1977) assumptions about motivational tendencies, however, are not completely transferable to a BCI context, as in his model, the tendency toward behavior or action taking is determined by the actor, who evaluates whether the valence of an action–outcome is high enough to justify taking action while considering the expectancies with which this outcome might be achieved. In a BCI setting, participants have already decided to participate in a study without extensive knowledge of BCI technology, so SOE cannot be reliably estimated. Consequently, AOE and OCE are more relevant in a BCI context compared with SOE, because the expectation of monetary reimbursement for participation is a clearer expectation than how a BCI system can be controlled.

Another important aspect to consider in the BCI context, when addressing the person–situation interaction, is the development of interest in the BCI task. Some participants may already have a strong interest in BCI technology and research, which serves as their primary motivation for participation. Others, at least initially, require external motivation, such as monetary reward. According to Rheinberg et al. (2000), such rewards represent incentives from future events, as they are received after completing the BCI experiment. However, as participants engage with the study, they acquire knowledge about BCI technology and its applications, which can increase their interest. This process reflects Hidi’s (2000) concept of situational interest, which may be initially driven by external incentives and may transform into individual interest, defined as a stable predisposition toward the activity itself (Ainley, 1998; Ainley et al., 2002; Renninger, 2000).

Interest and intrinsic motivation are closely linked: intrinsic motivation is generally described as motivation directed toward behaviors that are inherently interesting to the individual (Hidi, 2000; Rheinberg et al., 2000; Deci and Ryan, 1985; Ryan and Deci, 2000a). Thus, interest can be considered a key factor in the development of motivation (Schiefele, 1999). In a BCI context, using the system can itself be understood as a behavior-specific incentive, particularly for patient populations. ALS patients, for example, often exhibit high individual interest in BCI technology, and participation may fulfill needs for autonomy, competence, and meaningful engagement.

Regarding SMR-BCIs, which rely on self-regulated learning, Heckhausen’s Cognitive Model of Motivation (Heckhausen, 1977) is particularly applicable. In this model, a perceived situation leads to action, which produces an intended outcome or goal, generating desired consequences. Expectations are more influential for SMR-BCIs than for P300 BCIs, as P300 control does not depend on self-regulated learning. If SMR control is not initially a motivating goal, SDT (Ryan and Deci, 2000a) suggests that offering external rewards can initiate self-regulated learning. Over time, this process may foster intrinsic motivation and the desire to master SMR control. Therefore, providing immediate feedback or reward for correct actions can support learning and improve performance in SMR-BCI tasks (see Figure 4).

### 2.6 How motivation may influence P300-BCI performance: Johnson’s Triarchic model of the P300 amplitude

As the P300 signal is an autonomously elicited response, motivation cannot directly influence this signal and its quality. However, variables that mediate the psychological state of feeling motivated and the elicitation of the P300 signal may be influenced by motivation.

In his Triarchic Model of the P300 amplitude, Johnson (1986) summarized the main factors influencing the occurrence and amplitude of the P300 signal. He postulated three main dimensions influencing the P300 signal: (1) subjective probability, (2) stimulus meaning, and (3) information transmission. Each of these dimensions can be divided into several subdimensions. Johnson (1986) postulated that P300 amplitude is dependent on unequal contributions from the main dimensions subjective probability (SP), stimulus meaning (SM), and information transmission (IT). Within the SM dimension, the subdimensions include task complexity, stimulus complexity, and stimulus value. In the context of motivation, the stimulus value subdimension is particularly relevant. The assumption that higher P300 amplitudes occur when the stimulus has specific value was demonstrated by Begleiter et al. (1983). In their experiment, the highest P300 amplitude occurred in response to the highest monetary reward. The value of the target stimulus was increased in the payoff condition for correct responses. In addition, stimulus intensity can increase stimulus value (Johnson, 1986), for example, by increasing sound volume or light intensity, as greater attention is allocated to such stimuli, resulting in higher P300 amplitudes (Polich et al., 1996; Squires et al., 1977).

Within the dimension of IT, the subdimension of attention appears most important. Johnson (1986) hypothesized that higher P300 amplitudes occur when a participant is attentive to the presented stimuli than when instructed to ignore them. This assumption was confirmed, for example, by Polich (1986), who instructed participants to either count the auditory target stimuli, ignore them, or read a text while ignoring them. The highest P300 amplitudes were observed when participants were instructed to count stimuli, indicating conscious focus on the target stimulus. Therefore, participant involvement in the task is reflected by P300 amplitude (Yeung and Sanfey, 2004).

When using a P300 BCI, participants are instructed to silently count the number of times a letter is intensified, thereby leading to high participant involvement and controlled processing. However, increasing motivation through monetary reward may not only positively influence the SM dimension but also attention, as an increased P300 amplitude with increased monetary reward has been demonstrated (e.g., Goldstein et al., 2006; Yeung and Sanfey, 2004; Wu and Zhou, 2009). Within the SM dimension, only the category of stimulus value would be influenced, as BCI users are promised monetary rewards for correct letter selections, which should increase stimulus value. Because participants are more interested in stimuli that directly influence their payoff, they may allocate more attention to these stimuli. For clinical end users, the influential aspect may instead be BCI control itself. Additionally, IT must be guaranteed in a BCI context, as stimuli that cannot be distinguished or unclear feedback may decrease motivation and performance (see Figure 4).

Using this general framework, the potential influences of motivation on BCI control have been specified. However, the framework can be further condensed and refined for P300 and SMR input signals while still providing a foundation for future research and empirical testing.

## 3 The MotiVE models

### 3.1 The MotiVE P300 model of BCI control

**Figure 5:**
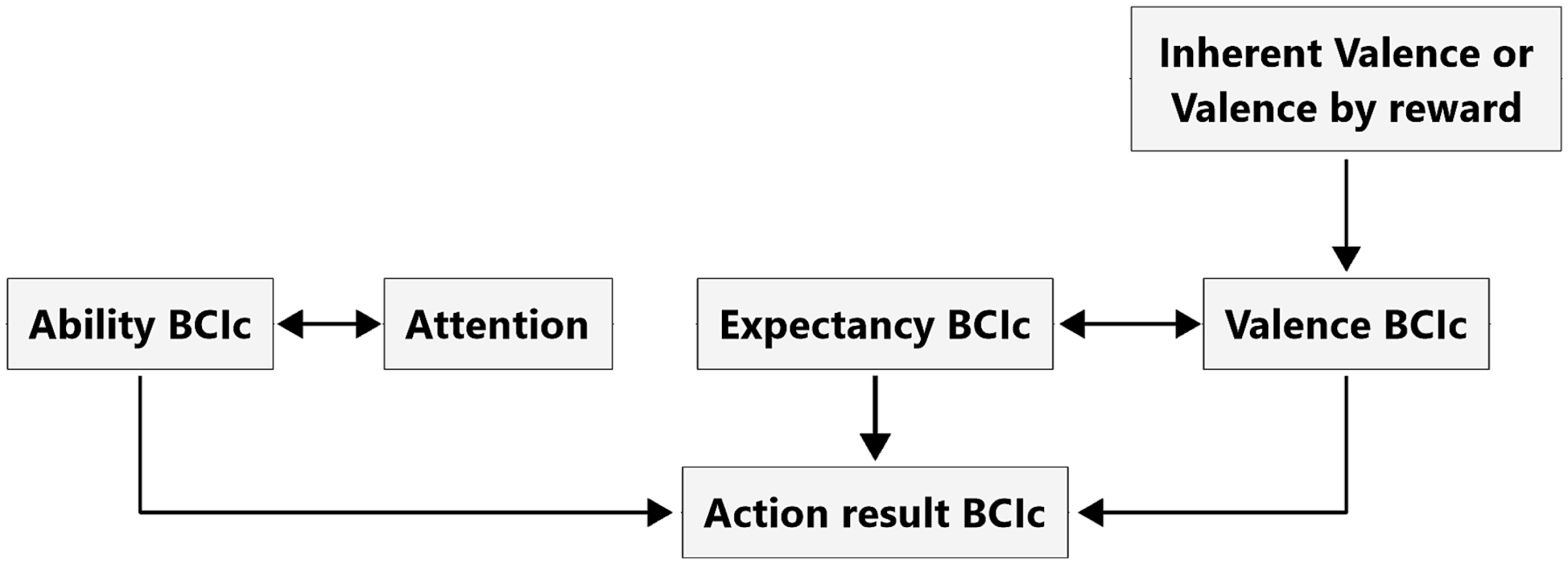
MotiVE P300 model. BCIc, BCI control.

BCIc, BCI control; att, attention; rbr, regulation of brain response; Vin, inherent valence; VbR, valence by reward; max, choice of maximum valence.

In the MotiVE P300 model, the original ability component was extended with an attention component. The attention component of Johnson’s Triarchic Model of P300 amplitude (Johnson, 1986) plays a major role in, and is a prerequisite for, successful P300 BCI use. However, ability for BCI use may still be influenced by situational components, such as equivocation of stimuli (Johnson, 1986) or, as pointed out by Kübler et al. (2011), brain anatomy. These influences (equivocation, brain anatomy) are summarized in this work in the ability dimension of the P300 Motivation model of BCI control including valence and expectancy (MotiVE). The multiplicative connection between the ability for BCI control and attention was chosen because the two components are interdependent. A change in the ability dimension (e.g., brain lesion, equivocation of signals, or similar influences) will directly influence attention, and, thus, the action result of BCI control (see Equation 6 and Figure 5).


$$\begin{array}{l} \text{Action result BCIc}=f(\text{ability BCIc}*\mathrm{att})*\\ \left[\sum_{j=1}^{n}(E\;\text{rbrBCIc}*\max(\mathrm{Vin},\mathrm{VbR})\right] \end{array}$$ (6)

To test the model mathematically, it is assumed that optimal BCI control corresponds to 50% accuracy. The other 50% accuracy are probably attributable to the technical part of the BCI, the input signal, the paradigm, the classification method and other technical aspects. Choosing 50% of accuracy for the human part and 50% for the technical part is somehow arbitrary at this point in time and probably must be adjusted by future work. Specifically the contribution of the subcomponents must be addressed for a final model including all aspects contributing to BCI performance. Based on previous findings (Käthner et al., 2013; Kleih et al., 2010), motivation may account for between 19 and 21% of the variance in BCI performance. For a conservative estimate, I assume a contribution of motivation of 10% of variance explanation. According to Equation 6, the remaining 40% of the variance would, therefore, be attributable to the ability to control a BCI (ability_BCIc = 20%) and the ability to sustain attention (att = 20%). An equal contribution of these two components is assumed in this work; however, this distribution requires empirical validation in future research. Because, in the proposed model, ability, attention, expectancy, and valence can reach a maximum value of 1, a rescaling of the equation components is required (Equation 7):


50%BCIaccuracy=(0.20x∗0.20x)∗0.10x (7)

This yields 50 = x^3^ * 0.20^2^ * 0.10. Solving for x gives x = $\sqrt[{3}]{50}÷0.20*0.20*0.10$ = 23,21. Substituting these values into the original equation results in the following approximation (Equation 8):


50=(4.64∗4.64)∗2.32 (8)

Consider a BCI user with a neurodegenerative disease who retains the general ability to operate a BCI (4.64) and is highly motivated (2.32), but whose capacity to sustain attention is limited to 75% of the duration expected in a healthy individual (4.64 × 0.75). According to the present model, the best achievable BCI accuracy in this case would be approximately 37%. Additionally to the potentially 50% reached by techical components, overall accuracy would then reach potentially 87%. In another scenario, a BCI user might be fully able to use the BCI (4.64) and sustain attention for as long as necessary (4.64) but shows reduced expectancy regarding whether the BCI will function effectively. Thus, expectancy would not be 1 but 0.7, yielding $\sqrt{2.32}*0.7*1*\sqrt{2.32}$ = 1.62 as a value for the motivation component. In total, a BCI accuracy of 4.64*4.64*1.62 = 34.87% could be reached for the human part of the system. Both scenarios represent only one of potentially many sessions for which an average would be calculated. It must be emphasized that the values presented in this work are purely illustrative and cannot currently be empirically validated. The proportion of variance attributable to motivation, BCI control ability, and attention requires further systematic investigation, as does the nature of the components themselves and their potential subcomponents. We do not know yet, to what extent a change in one component influences the other components or whether they are influenced as proposed here. Also, the influence of the technical part of the BCI system which might add up to another 50% accuracy in the best possible case must be considered. The calculations presented in this work are intended solely to demonstrate how the model can be applied mathematically.

### 3.2 The MotiVE SMR model of BCI control

In the MotiVE SMR model, the attention component was, in addition to its traditional role in attention allocation, interpreted as the user’s ability to realize contingencies between actions and consequences during learning, or, in other words, the user’s susceptibility to operant learning (OLe). Again, possible personal (e.g., brain lesions) and situational components (equivocation in IT; Johnson, 1986) were included in the model within the ability component (see Equation 9):


$$\begin{array}{l} \text{Action result BCIc}=f(\text{abilityBCIc}*\mathrm{OLe})*\\ \left[\sum_{j=1}^{n}(E\;\text{rbrBCIc}*\max(\mathrm{Vin},\mathrm{VbR})\right] \end{array}$$ (9)

The MotiVE SMR model. BCIc, BCI control; OLe, operant learning; rbr, regulation of brain response; Vin, inherent valence; VbR, valence by reward; max, choice of maximum valence.

For the MotiVE SMR model, valence was presumed to follow equivalent rules as in P300-BCI. The expectancy component appears to be more important compared to the P300-BCI, as the expectation for being able to regulate brain responses may support learning (Vollmeyer and Rheinberg, 2000). Therefore, Heckhausen’s (1977) model is used to account for the expectancy component in a BCI context. First, situation–outcome expectancy is required, in which a BCI user realizes that the situation of using an EEG system and being instructed how to imagine movement will lead to BCI control. Situation–outcome expectancy is a prerequisite before an action can begin. Although initiating an action, which in this case would be motor imagery, action–outcome expectancy needs to be developed so that the BCI user believes that motor imagery leads to BCI control. Then, in the final step, outcome–consequence expectancy emerges, ensuring that motor imagery (action) is maintained, leading to BCI control (outcome), which in turn leads to the consequence of either receiving monetary reward (extrinsic) or being able to use a BCI for communication (intrinsic). In the MotiVE SMR model, therefore, the expectancy component (E) for successful regulation of brain response (rbr) and subsequent BCI control (BCIc) consists of two of the three mentioned expectancies postulated by Heckhausen (1977): (1) AOE and (2) OCE. Therefore, the MotiVE SMR model presented in Equation 10 is suggested.


$$\begin{array}{l} \text{Action result BCIc}=f(\text{abilityBCIc}*\mathrm{OLe})*\\ \left[\sum_{j=1}^{n}((\mathrm{AOE}*\mathrm{OCE})*\max(\mathrm{Vin},\mathrm{VbR})\right] \end{array}$$ (10)

The MotiVE SMR model. BCIc, BCI control; OLe, operant learning; AOE, action–outcome expectancy; OCE, outcome–consequence expectancy; Vin, inherent valence; VbR, valence by reward.

**Figure 6:**
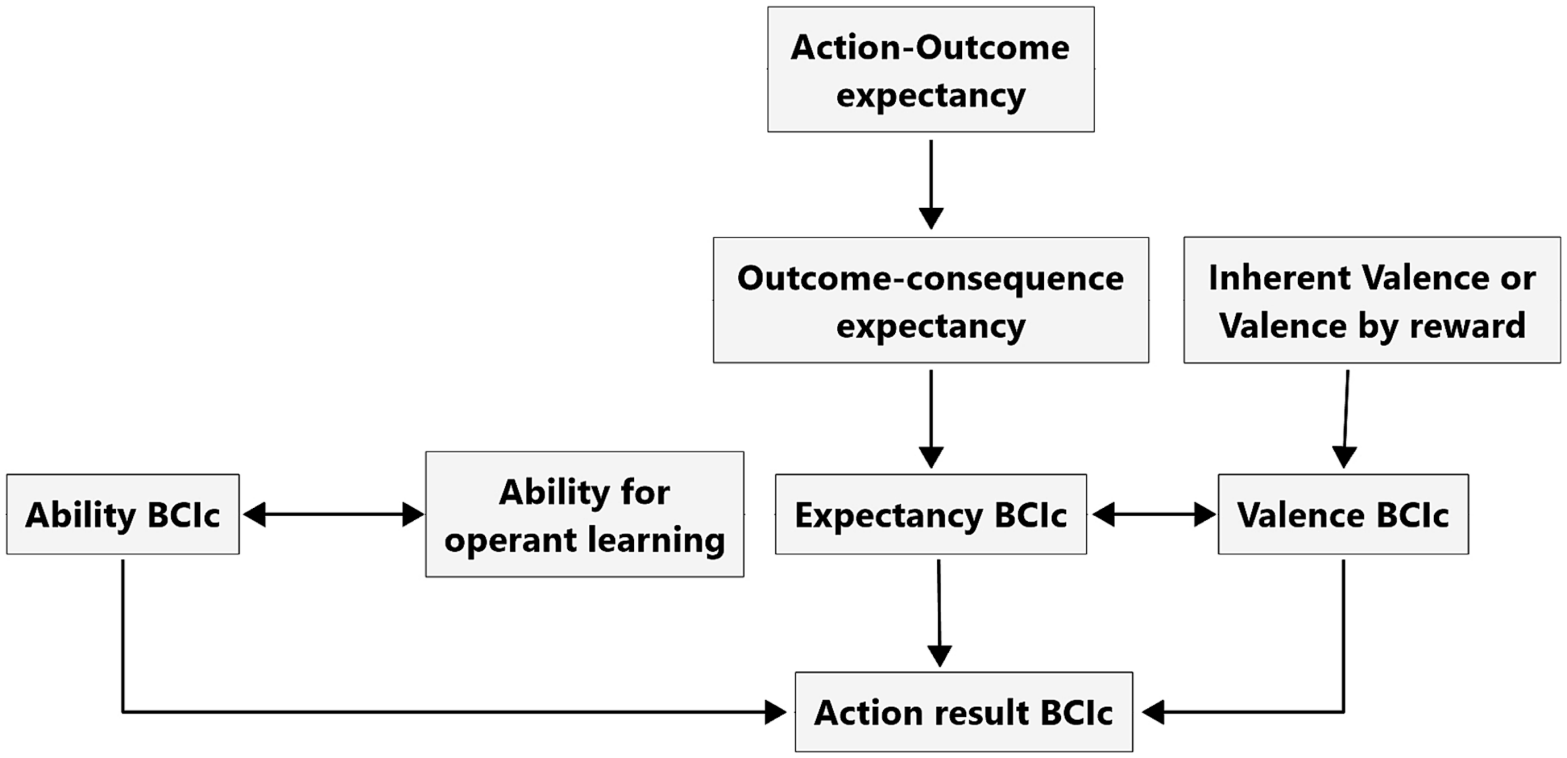
MotiVE SMR model of BCI control. BCIc, BCI control; AOE, action–outcome expectancy; OCE, outcome–consequence expectancy; Vin, inherent valence; VbR, valence caused by reward.

The multiplicative relationship between AOE and OCE was chosen because these expectancies are interdependent. An outcome can only lead to a consequence if the corresponding action produces that outcome. In the BCI context, users recognize this connection and understand that successful BCI control depends on their ability to regulate SMR. The resulting MotiVE SMR model is illustrated in Figure 6.

Mathematically, the same parameter values proposed for the P300 MotiVE model could be used provisionally until more precise data are available regarding the variance explained by the individual model components. For both models, mathematical operations may require refinement as new evidence emerges from future BCI studies. The weighting of the individual components may also change in subsequent versions, as the models presented in this study represent an initial *theoretical* framework.

## 4 Discussion

Although the importance of addressing the human factor in the BCI loop has been emphasized (Kübler et al., 2011; Valeriani and Vaughan, 2025), interdisciplinary models remain rare (Jeunet et al., 2017). Regarding motivation, this study summarizes primarily psychological theories that can be applied to the BCI context and may provide useful insights for understanding user behavior. The two MotiVE models, the MotiVE P300 and MotiVE SMR models, were presented in this study as a synthesis of existing BCI literature and motivation theory. The theoretical basis for both motivation models is expectancy × value theories (Feather, 1982).

### 4.1 Expectancy in the MotiVE models

In the MotiVE models, expectancy refers to a person’s belief that they can successfully perform a task. In brain–computer interface (BCI) research, this is closely related to mastery confidence, which represents the user’s confidence in their ability to control the BCI. Previous studies (Nijboer et al., 2010; Kleih and Kübler, 2014; Kober et al., 2016) suggest that mastery confidence is a key predictor of BCI performance. However, to fully understand expectancy’s role, it needs to be manipulated experimentally in future research. By deliberately increasing expectancy, for example, by providing encouraging instructions or positive feedback, participants’ motivation should increase if the underlying theoretical assumptions are correct. Conversely, decreasing mastery confidence is expected to reduce motivation and impair BCI performance.

### 4.2 Valence in the MotiVE models

The classification of valence quality depends on a person’s position along the continuum from extrinsic to intrinsic motivation. As such, the interpretation of the MotiVE models should be grounded in SDT (Ryan and Deci, 2000a) and CET (Deci and Ryan, 1985). As with the expectancy component, further research is needed to investigate the valence component by manipulating it, not only through monetary reward. As pointed out by Reeve (2008), when actions are unattractive, rewards may be required to initiate or sustain a given action. Indeed, an attempt was made to recruit participants for a BCI study at the University of Würzburg from April to June 2012 without offering any reward for participation, in order to assess the reward-independent attractiveness of BCI studies in the local student population. Only one volunteer contacted the research team, and she did not attend the appointment after being informed that the absence of monetary compensation was not a typographical error in the recruitment notice.

The implementation of social feedback stimuli (e.g., the presentation of images of specific individuals) or even artificially created faces for learning support (Pillette et al., 2020) might increase valence for some participants but not for others. However, standardized versus individualized approaches should be further investigated. Additionally, future research needs to determine whether it is possible to identify the spectrum of motivation from external regulation to integrated regulation (Ryan and Deci, 2000a) through experimental manipulation (Kleih and Kübler, 2013).

#### 4.2.1 The value of valence

It is proposed that valence in a BCI context cannot be negative. Negative valence would imply that a BCI user prefers to avoid the outcomes of BCI control. However, participation in BCI research is voluntary; if a person experiences negative valence, they would simply not participate. Therefore, in this context, valence ranges from 0 to 1.

Moreover, inherent valence ( $V_{\text{in}}$ ) and valence by reward ( $V_{\mathrm{bR}}$ ) are likely independent. For example, a reward-sensitive healthy participant could have $V_{\text{in}}=0\;$ while $V_{\mathrm{bR}}\;$ is at maximum, whereas for an ALS patient, the opposite is more probable. Based on this reasoning, this study proposes focusing on the dominant valence as the primary driver of behavior and disregarding the less distinct valence quality, which contributes minimally, if at all, to overall motivation. Mathematically, this is expressed as follows:


$$\text{Valence}=\max(V_{\text{in}},V_{\mathrm{bR}})$$

One might wonder why the two valence components were not connected multiplicatively, given evidence that extrinsic rewards can undermine intrinsic motivation (e.g., overjustification effects; Lepper et al., 1973). However, even if inherent valence decreases due to external rewards, one valence quality remains dominant and primarily guides behavior. Multiplication would also falsely imply interdependence between $V_{\text{in}}$ and $V_{\mathrm{bR}}$ , which is not the case, as one valence component can change independently of the other. Additionally, if one valence component is maximal and the other is zero, multiplication would reduce the overall value to zero, which would misrepresent the true motivational strength.

Combining the two valence components additively is also problematic. $V_{\text{in}}\;$ and $V_{\mathrm{bR}}$ values range from 0 to 1, but both belong to the same construct of valence. Addition would incorrectly suggest that maximal motivation requires both valences to be maximal. In practice, highly motivated ALS patients or healthy participants motivated by a monetary reward may achieve maximal motivation even when only one valence component is strong.

In summary, the dominant valence component, that is, the one with the higher value, best represents overall motivational strength and should be used to calculate the motivation estimate.

### 4.3 The person–situation interaction

I propose that the interaction between the person and the situation influences the motivation for BCI control (see Figure 4). Therefore, the environmental context in which BCI use takes place must be considered (Horowitz et al., 2021). Relatives, friends, researchers (Branco et al., 2023), and caregivers (Taherian and Davies, 2018; Schicktanz et al., 2015) may hold expectations regarding BCI use, and these expectations can shape the user’s subjective expectancy (Padfield et al., 2024), perceived value, and consequently, their motivation. In some cases, these expectations may even conflict with one another: while caregivers may hope that BCI use proceeds quickly and smoothly, relatives may prefer longer sessions that allow for troubleshooting and the establishment of communication or environmental control.

Results obtained in a laboratory environment cannot be transferred directly to a home-use environment in BCI. For some participants, feeling comfortable in their own apartment might increase motivation for BCI use, while for others, researchers might be perceived as intruders in private space. Both experiences may influence the valence of BCI control.

Additionally, the interaction between a BCI researcher and the BCI user is a relationship that might be investigated in its own right (Roc et al., 2019). Is a BCI researcher more likely to be perceived as medical personnel or as an acquaintance? Another potential factor to be investigated is trust. It is possible that the feeling of trust toward a BCI professional who explains BCI use for the first time may influence a BCI user’s individual expectancy.

### 4.4 Additional variables potentially affecting BCI use

Among other potential influencing factors on motivation that need to be investigated are cognitive dissonance (Festinger, 1957), self-efficacy beliefs (Bandura, 1977), interest (Hidi, 2000), and the neurobiological basis reflected in individual differences in behavioral inhibition and behavioral activation (Carver and White, 1994).

For some participants, there may be a discrepancy between reported motivation and actual motivation that cannot be consciously reported (Festinger, 1957). Participants who are not rewarded may unconsciously justify their participation as worthwhile and, therefore, report high motivation. Thus, cognitive dissonance could explain the fact that most reports by rewarded and non-rewarded participants often do not differ (Festinger, 1957). It is, however, not uncommon that behavioral measures differ from cognition reports (Ploger et al., 2021). Dissonance reduction as a cognitive process might require cognitive resources that could decrease the ability to use a BCI (Equations 6, 10). The possible influence of cognitive dissonance should, therefore, be investigated in future empirical studies.

Bearing in mind the preceding discussion on expectancy and its role in BCI performance, self-efficacy beliefs should also be considered as a potential influencing factor (Bandura, 1977, 1997). Self-efficacy refers to a person’s conviction that they can perform a specific action, rather than a general judgment of their personality or abilities (Bandura, 1977). In other words, it represents a person’s expectation of success or belief in their ability to master a task at a certain performance level. This is particularly relevant for SMR-BCI control, as SMR control is a self-regulatory task (Heckhausen, 1977) for which mastery confidence, or expectancy, has a stronger influence on performance than in P300-BCI tasks.

Research suggests a clear link between self-efficacy beliefs and academic achievement (e.g., Lent et al., 1984; Zimmerman, 1990, 2000). Of particular interest is the observation that individuals with high self-efficacy tend to set more specific goals, whereas those with low self-efficacy set vague goals, which complicates the evaluation of progress (Cleary and Zimmerman, 2001, 2004). In the context of SMR-BCI, users with high self-efficacy may set trial-based goals (e.g., steering a cursor into a target area), while other users may adopt vague goals (e.g., being successful in many trials). Goal evaluation is more straightforward in the former case, especially when performing, for example, SMR-BCI sessions consisting of 10 blocks of 4 runs, each containing 25 trials. Furthermore, students with high self-efficacy have been shown to be more persistent even in challenging tasks (Multon et al., 1991), which may support SMR-BCI performance, as participants often need time to discover effective strategies. High self-efficacy is also associated with greater use of self-regulated learning strategies (Schunk and Zimmerman, 1997) and a tendency to attribute success internally. This links directly to SDT (Ryan and Deci, 2000a, 2000b), as an internal locus of control enhances motivation, which may, in turn, improve BCI performance (Burde and Blankertz, 2006). These variables might influence the ability component in BCI use but also the expectancy to be able to successfully use a BCI (see Equations 6, 10). Therefore, future studies should investigate the influence of self-efficacy beliefs on BCI performance, particularly in SMR-BCI tasks where self-regulation, goal setting, and expectancy of success are critical.

Another psychological construct that may be applicable to this context is the sensitivity to rewards (Gray, 1972, 1987; Gray and McNaughton, 2000). In some healthy participants, the rewards provided for study participation may not be perceived as rewarding, or participants may be less sensitive to rewards. In these individuals, the Behavioral Inhibition System (BIS) is more dominant, which is associated with higher right prefrontal cortex activation, heightened sensitivity to punishment, and avoidance-oriented behavior. Conversely, participants who are highly sensitive to rewards tend to have a dominant Behavioral Activation System (BAS; Carver and White, 1994; Corr, 2016). Individuals with a more active BAS typically show higher left prefrontal activation, increased reward sensitivity, and more approach-oriented behavior. Consequently, experimental designs in BCI research that aim to influence motivation through rewards should consider controlling for this neurobiological predisposition. Within the MotiVE models, BIS/BAS orientation might influence the ability component in the mathematical model (see Equations 6, 10). Reward salience and its effects on performance could be adjusted according to the individual’s BIS/BAS orientation. For BIS-oriented individuals, the inclusion of multimodal performance feedback, such as a pleasant tone combined with a visual indicator (e.g., a smiley), might facilitate learning and enhance motivation or may alternatively be overwhelming and decrease motivation. Indeed, gamified feedback (Kober et al., 2024) could be adjusted for both BIS and BAS orientations.

### 4.5 The mathematical model

The mathematical models proposed in this study (see Equations 6, 10) should be rigorously tested in future research. Although previous work has suggested that motivation may explain approximately 19% of the variance (Käthner et al., 2013), this was not the primary focus of past investigations. Whether motivation and its contribution to variance can be reliably captured in a mathematical formula remains an open question. Additionally, the mathematical interconnection between the components proposed in this study to contribute to motivation must demonstrate its practical usefulness through empirical validation.

### 4.6 Limitations

The MotiVE P300 and MotiVE SMR models represent streamlined versions of a theoretical framework (see Figure 6). Future iterations may require the addition, removal, or modification of certain components, for example, incorporating the influence of social context as a more prominent factor. In this study, only P300- and SMR-based BCIs were considered, despite the existence of other BCI input signals (Valeriani and Vaughan, 2025). Although it may be plausible to extend these models to other input modalities, such as steady-state visual evoked potential (SSVEP)-based BCIs, certain components may gain relative importance depending on the specific input signal, for example, environmental factors such as distraction. Importantly, both models have not been empirically tested but have been derived from existing literature and established psychological theory. The practical utility of this approach remains an open question for future research.

## 5 Conclusion

Both MotiVE models presented in this study appear to offer better predictions of the influence of motivation on BCI performance than existing motivation models that were not specifically developed for the BCI context. However, the MotiVE models, as well as the general theoretical framework introduced in this work, should be tested in experimental settings to evaluate their usefulness and added value.

## Data Availability

The original contributions presented in the study are included in the article/supplementary material, further inquiries can be directed to the corresponding author.
